# Video-Based Educational Interventions for Patients With Chronic Illnesses: Systematic Review

**DOI:** 10.2196/41092

**Published:** 2023-07-19

**Authors:** Nikita Deshpande, Meng Wu, Colleen Kelly, Nicole Woodrick, Debra A Werner, Anna Volerman, Valerie G Press

**Affiliations:** 1 Pritzker School of Medicine University of Chicago Chicago, IL United States; 2 Section of General Internal Medicine Department of Medicine University of Chicago Chicago, IL United States; 3 Department of Pediatrics Boston Children's Hospital Boston, MA United States; 4 Corporate Engagement & Strategic Partnerships Arizona State University Tempe, AZ United States; 5 The University of Chicago Library Chicago, IL United States

**Keywords:** chronic disease, patient education, video-based interventions, video education, technology, health literacy, self-management

## Abstract

**Background:**

With rising time constraints, health care professionals increasingly depend on technology to provide health advice and teach patients how to manage chronic disease. The effectiveness of video-based tools in improving knowledge, health behaviors, disease severity, and health care use for patients with major chronic illnesses is not well understood.

**Objective:**

The aim of this study was to assess the current literature regarding the efficacy of video-based educational tools for patients in improving process and outcome measures across several chronic illnesses.

**Methods:**

A systematic review was conducted using CINAHL and PubMed with predefined search terms. The search included studies published through October 2021. The eligible studies were intervention studies of video-based self-management patient education for an adult patient population with the following chronic health conditions: asthma, chronic kidney disease, chronic obstructive pulmonary disease, chronic pain syndromes, diabetes, heart failure, HIV infection, hypertension, inflammatory bowel disease, and rheumatologic disorders. The eligible papers underwent full extraction of study characteristics, study design, sample demographics, and results. Bias was assessed with the Cochrane risk-of-bias tools. Summary statistics were synthesized in Stata SE (StataCorp LLC). Data reporting was conducted per the PRISMA (Preferred Reporting Items for Systematic Reviews and Meta-Analyses) checklist.

**Results:**

Of the 112 studies fully extracted, 59 (52.7%) were deemed eligible for inclusion in this review. The majority of the included papers were superiority randomized controlled trials (RCTs; 39/59, 66%), with fewer pre-post studies (13/59, 22%) and noninferiority RCTs (7/59, 12%). The most represented conditions of interest were obstructive lung disease (18/59, 31%), diabetes (11/59, 19%), and heart failure (9/59, 15%). The plurality (28/59, 47%) of video-based interventions only occurred once and occurred alongside adjunct interventions that included printed materials, in-person counseling, and interactive modules. The most frequently studied outcomes were disease severity, health behavior, and patient knowledge. Video-based tools were the most effective in improving patient knowledge (30/40, 75%). Approximately half reported health behavior (21/38, 56%) and patient self-efficacy (12/23, 52%) outcomes were improved by video-based tools, and a minority of health care use (11/28, 39%) and disease severity (23/69, 33%) outcomes were improved by video-based tools. In total, 48% (22/46) of the superiority and noninferiority RCTs and 54% (7/13) of the pre-post trials had moderate or high risk of bias.

**Conclusions:**

There is robust evidence that video-based tools can improve patient knowledge across several chronic illnesses. These tools less consistently improve disease severity and health care use outcomes. Additional study is needed to identify features that maximize the efficacy of video-based interventions for patients across the spectrum of digital competencies to ensure optimized and equitable patient education and outcomes.

## Introduction

### Patient Education and Health Outcomes

Patients’ chronic illness management skills, including medication use, symptom monitoring, and other health behaviors, are a vital step in achieving good health outcomes [1-3]. Effective self-management of chronic illnesses relies on several factors, including possessing knowledge about one’s condition, believing that one’s behaviors affect one’s health, and having the ability to use and access to necessary resources to execute recommended health behaviors [4]. Theories guiding patient education include adult learning theory, which emphasizes self-directedness in learning, the incorporation of prior knowledge, and problem-centered approaches [5]. In addition, self-efficacy theory emphasizes patients’ belief in their power to manage their disease [6]. Patient education can directly affect knowledge and self-efficacy [7,8]; for example, comprehensive in-person patient education has been shown to improve quality of life (QOL), reduce hospitalization, and reduce readmission among patients with heart failure [9]. In addition to improving patient health outcomes, adequate chronic illness management can reduce health care costs; for example, an estimated US $100 billion of health care costs in the United States could have been avoided in 1 year through improved medication adherence [10].

### Technology and Patient Education

Although patient education is often highly valued by health care professionals, the lack of time and insufficient staffing are cited as barriers to providing optimal in-person patient education, if it is provided at all [11-14]. The average primary care visit involves only 16 minutes of face-to-face time between clinician and patient [15]. Because of these constraints, patient education interventions that can be used outside of the clinic, including video-based resources, SMS text message reminders, and interactive modules have been developed to replace or augment in-person patient counseling. Digital therapeutics (eg, mobile apps) have been shown to improve outcomes in diabetes and smoking cessation [16-18]. Technology-based resources may be less effective for some patients, such as those without access to technology devices or the internet or those with low eHealth literacy but could be tailored to individual factors such as health literacy, eHealth literacy, and self-efficacy [19-22].

### Patient Education and the COVID-19 Pandemic

In addition to the growing interest in technology-based interventions more broadly, there is increased pressure in the wake of the COVID-19 pandemic to develop and implement virtual care and education resources for patients because clinics halted in-person visits during the pandemic [23,24]. Stay-at-home orders and physical distancing led to virtual patient programming by primary care clinics, geriatricians, and pharmacists [25-27]. This drastic change in health care delivery yielded substantial literature studying its effects on patient satisfaction, behaviors, and health outcomes [28]. Studies of digital patient education tools for patients with cancer and fertility issues found that such tools resulted in increased health knowledge but were not sufficient in terms of addressing patients’ emotional needs and were associated with patient stress and disappointment [29,30]. As the COVID-19 pandemic evolves, provider time constraints persist, and technology-based interventions continue to expand more broadly, finding ways to optimize patient education virtually will continue to be a priority.

### Efficacy of Video-Based Education Tools

Digital interventions are tools that use technology to improve patients’ health and include apps, games, modules, and videos. Video-based educational interventions, which are a form of digital intervention, not only use educational principles such as adult learning theory and self-efficacy theory but also navigate challenges of disparities in technology access and eHealth literacy. The efficacy of digital interventions, including video-based education tools, has been studied for specific chronic illnesses, but, to our knowledge, a systematic review of the efficacy of video interventions across major chronic illnesses has not been performed. In addition, studies of the qualities that make educational content most effective, including setting, frequency, and intervention components, have been inconclusive [31]. A more complete understanding of the efficacy of video-based education tools for chronic illnesses can meaningfully guide the development of new patient education interventions. Therefore, this study aimed to systematically review the literature to determine whether patient-targeted video-based educational self-management tools for chronic illnesses are effective interventions to improve self-management and health outcomes.

## Methods

### Search Strategy

We conducted a search of the English literature in consultation with a medical librarian (DW). Our initial search was conducted in MEDLINE using Medical Subject Headings (MeSH) terms related to chronic illness combined with terms to identify intervention studies, which included *video technology*, *self-management*, and *hospital discharge*, ultimately yielding the final search terms (Multimedia Appendix 1). We conducted parallel searches in PubMed and CINAHL. Searches were not restricted to specific publication years and included studies published through October 2021.

### Inclusion and Exclusion Criteria

The inclusion criteria were as follows: (1) adult patient population (aged ≥18 years); (2) intervention studies of video-based self-management patient education; and (3) one of the following chronic health conditions: asthma, chronic kidney disease (CKD), chronic obstructive pulmonary disease (COPD), chronic pain syndromes, diabetes, heart failure, HIV infection, hypertension, inflammatory bowel disease (IBD), and rheumatologic disorders. Articles with diverse digital interventions were included so long as at least 1 component was video based. Video-based self-management patient education interventions encompassed videos that aimed to educate patients regarding their disease or its management. Articles were excluded if they focused on chronic conditions other than those outlined in the inclusion criteria (eg, malignancy, other infectious diseases, injury or trauma, surgery, intellectual disabilities, prediabetes, obesity, ostomy care, and chronic psychiatric conditions), had pediatric or clinician study populations, were noninterventional studies, or involved interventions that were not video based or not designed for patients.

### Article Selection

The articles identified from all electronic databases were evaluated for duplicates to ensure that each was a unique manuscript. Next, title and abstract reviews were conducted to evaluate for inclusion. Papers not clearly excluded through title and abstract review underwent article extraction review. Of the 112 papers, 10 (9%) were reviewed initially by the study team to ensure consensus and validate the extraction form. Data from the articles were extracted into a standardized REDCap (Research Electronic Data Capture; Vanderbilt University) form by an author (VP, CK, MW, AV, and ND), and 19.6% (22/112) of the articles underwent a second independent extraction by a different author (VP, CK, MW, and AV) for ongoing quality assurance. The standardized form prompted extraction of the following elements: study characteristics (location, enrollment, and size), study design (randomized controlled trial [RCT] vs pre-post study and intervention components), sample demographics, and results.

### Data Analysis and Synthesis

**Figure 1:**
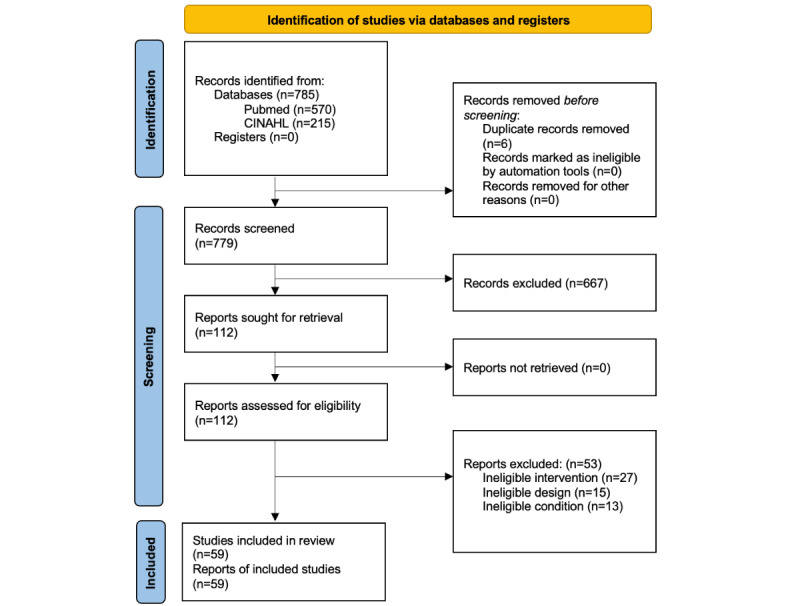
Identification of studies via databases and registers.

Articles were qualitatively evaluated to determine heterogeneity across key extraction components, including study characteristics, study design, sample demographics, and results. After the qualitative evaluation, data were quantified and summarized across these same components. Data visualization and descriptive statistics were generated using Stata SE (version 15.1; StataCorp LLC). Meta-analysis was not possible because the program outcomes were too heterogeneous. Data reporting was conducted per the PRISMA (Preferred Reporting Items for Systematic Reviews and Meta-Analyses) checklist (Figure 1 [32]).

### Quality and Bias Assessment

Risk of bias was assessed by applying the revised Cochrane risk-of-bias tool for randomized trials (RoB 2) for the included RCTs and the Risk of Bias in Nonrandomized Studies of Interventions (ROBINS-I; The Cochrane Collaboration) tool for the included pre-post studies [33,34]. The RoB 2 assessed multiple domains, including risk of bias from the randomization process, deviations from the intended intervention, missing data, measurement of outcome, and selection of reported results [33]. The ROBINS-I tool also assessed multiple domains, including risk of bias from confounding, selection of study participants, classification of the intervention, deviations from intended interventions, missing data, outcome measurement, and selection of reported results [34].

## Results

### Initial Search and Title and Abstract Review

Of the 785 studies that underwent title and abstract review, 112 (14.3%) were eligible for full article extraction (Figure 1). Of the 112 articles extracted, 59 (52.7%) were eligible for inclusion in this review (Table 1). Studies were excluded if they did not include an intervention (27/112, 24.1%), were pilot or qualitative studies (15/112, 13.4%), did not include eligible conditions (13/112, 11.6%), or did not use a video-based self-management intervention (3/112, 2.7%).

**Table 1:** Extracted studies: design, disease, intervention, outcomes, and results (n=59).

Studies	Design	Setting	Participants, n	Disease of interest	Components of intervention	Length of follow-up	Outcomes	Results
Albert et al [35], 2007	RCT^a^	Inpatient and home	76	HF^b^	Comparison group: printed materials and in-person counseling; treatment group: educational video, printed materials, and in-person counseling	90 days	Health care use, disease severity, and health behavior	No substantial effects on HF hospitalization and emergency care after intervention or discharge; significant increase in HF-related telephone advice (*P*=.04), decrease in number of symptoms from baseline (*P*=.04), and increase in score on HF self-care measure (*P*=.01)
Albikawi et al [36], 2016	RCT	Outpatient	159	Diabetes	Comparison group: in-person counseling; treatment group: educational video, printed materials, in-person counseling, and telephone or video counseling	2 weeks and 3 months	QOL^c^	No substantial decrease in anxiety and depression at 2 weeks; decrease in anxiety and depression at 3 months (*P*<.001)
Allam et al [37], 2015	RCT	Home	155	Rheum conditions	Comparison group: none; treatment group: educational video	2 months and 4 months	Health behavior, health care use, medication use, and self-confidence	No effects on exercise behavior, health care use, prescription medication use, and patient self-empowerment
Barker et al [38], 2020	RCT	Inpatient	196	COPD^d^	Comparison group: printed materials; treatment group: educational video and printed materials	1 month and 3 months	Health care use	Intervention did not increase pulmonary rehabilitation uptake
Bell et al [39], 2012	RCT	Home	64	Diabetes	Comparison group: none; treatment group: educational video	3, 6, 9, and 12 months	Disease severity	Significantly lower HbA_1c_^e^ level at 3 months (*P*=.02), increased instances of hypoglycemia (*P*=.05), no effects on HbA_1c_ level at 6 to 12 months, and no effects on frequency of hyperglycemic episodes
Boulware et al [40], 2013	RCT	Outpatient	130	CKD^f^	Comparison group: in-person counseling; treatment group: educational video, printed materials, and in-person counseling	6 months	Health behavior	Substantial increase in kidney donation discussion with physician; no effects on kidney donation discussion with family
Boyde et al [41], 2018	RCT	Outpatient and home	171	HF	Comparison group: printed materials and in-person counseling; treatment group: educational video, printed materials, and in-person counseling	28 days, 3 months, and 12 months	Health care use	No effects on HF-related unplanned hospital readmission, HF knowledge, and self-care; decrease in all-cause hospital readmission at 12 months (*P*=.005)
Calderon et al [42], 2009	RCT	ED^g^	128	HIV infection	Comparison group: in-person counseling; treatment group: educational video	Immediately after the intervention	Knowledge	Noninferior changes in score on HIV infection knowledge assessment
Chen et al [43], 2010	RCT	Outpatient	60	Asthma	Comparison group: in-person counseling; treatment group: educational video, printed materials, and telephone or video counseling	1 month	Medication use, health behavior, health care use, and self-confidence	Significant increase in self-reported medication adherence (*P*=.008), self-monitoring behavior (*P*=.001), environmental control and avoidance of triggers behavior score (*P*<.001), exercise behavior score (*P*=.02), self-efficacy in prevention of asthma attacks (*P*=.03), and self-efficacy of managing an asthma attack (*P*=.02)
Cordina et al [44], 2001	RCT	Home and other	119	Asthma	Comparison group: none; treatment group: educational video, printed materials, and in-person counseling	4, 8, and 12 months	QOL, disease severity, health behavior, medication use, and health care use	No difference in health-related QOL, PEF^h^, and self-reported inhaler compliance; significant increase in perceived vitality (*P*=.001), improved inhaler technique (*P*=.02), and decreased self-reported hospitalization (*P*=.002)
Dilles et al [45], 2011	RCT	Inpatient	30	HF	Comparison group: printed materials and in-person counseling; treatment group: educational video, interactive module, and quiz-based teaching	3 months	Health behavior and knowledge	No substantial effects on self-reported knowledge of HF, treatment, symptoms, and recognition of symptoms; no substantial effects on patient engagement in self-care behaviors
Ebrahimabadi et al [46], 2018	RCT	Outpatient	80	Asthma	Comparison group: printed materials and in-person counseling; treatment group: educational video	1 month	Medication use	Educational video was inferior to printed materials for medication adherence (*P*<.05)
Elander et al [47], 2011	RCT	Home	108	Chronic pain syndromes	Comparison group: printed materials; treatment group: educational video and printed materials	6 months	Health behavior and QOL	Significant increase in patient intention and motivation to self-manage pain (*P*<.001); no substantial changes in frequency of use of pain coping strategies
Emmett et al [48], 2005	RCT	NR^i^	217	Hypertension	Comparison group: none; treatment group: educational video, printed materials, and other decision aid	14 days and 3 years	Disease severity	No substantial difference in BP^j^, mean 10-year CVD^k^ risk, and consultations per year
Gerber et al [49], 2005	RCT	Outpatient	183	Diabetes	Comparison group: none; treatment group: educational video, interactive module, and quiz-based teaching	1 year	Disease severity and knowledge	No substantial effects on HbA_1c_ level, BP, BMI, self-efficacy, and diabetes knowledge; significant improvement in HbA_1c_ level among patients with lowest health literacy (*P*=.04); and increase in perceived susceptibility to diabetes complications (*P*=.009)
Glasgow et al [50], 1996	RCT	Home and outpatient	94	Diabetes	Comparison group: in-person counseling; treatment group: educational video, interactive module, printed materials, in-person counseling, telephone or video counseling, and nutritional counseling	3 months	Health behavior, disease severity, and QOL	Significant decrease in fat-related dietary habit questionnaire (*P*<.001); decrease in serum cholesterol level (*P*<.001); no substantial change in HbA_1c_ level and QOL
Glasgow et al [51], 2009	RCT	Home and outpatient	155	Diabetes	Comparison group: in-person counseling, printed materials, nutritional counseling, and group discussion; treatment group: educational video and interactive module	6 months	Health behavior, medication use, self-confidence, and disease severity	Noninferiority for changes in healthy eating days, physical activity days, medication adherence, self-efficacy, HbA_1c_ level, LDL^l^ cholesterol level, and systolic BP
Gravely et al [52], 2011	RCT	Inpatient	23	Diabetes	Comparison group: printed materials; treatment group: educational video	Day of discharge	Knowledge	A quantitative but not substantial change in score on diabetic foot care test
Hickman et al [53], 2015	RCT	Home	116	Hypertension	Comparison group: game; treatment group: educational video	4 months	Disease severity	No substantial difference in BP between educational video and game; substantial improvements in BP after both interventions
Houston et al [54], 2017	RCT	Home and outpatient	527	Hypertension	Comparison group: educational video (traditional); treatment group: educational video (storytelling)	6 months	Disease severity	No difference in systolic and diastolic BP as well as medication adherence between traditional and storytelling videos
Huang et al [55], 2008	RCT	Home and outpatient	148	Asthma	Comparison group: in-person counseling, printed materials, and telephone or video counseling; treatment group: educational video	6 months	Health behavior, self-confidence, disease severity, health care use, and knowledge	In-person counseling had a significantly larger increase in knowledge related to asthma self-care (*P*<.001), assessment of asthma self-care behavior (*P*<.001), self-efficacy (*P*<.001), and FEV_1_^m^ prebronchodilation (*P*=.02) than the educational video; no substantial difference in unscheduled health care use and medication dosage
Jerjes et al [56] 2007	RCT	Outpatient	41	Chronic pain syndromes	Comparison group: educational video; treatment group: educational video and relaxation tapes	6 weeks	Disease severity	No substantial improvement in level of disability, BDI^n^ score, or self-efficacy 6 weeks reported after the intervention
Kamat et al [57], 2018	RCT	Outpatient	84	IBD^o^	Comparison group: none; treatment group: educational video	6 months and 12 months	QOL, medication use, health care use	Significant 12-month decrease in self-reported depression (*P*<.04); significant 6- and 12-month increase in follow-up visit compliance (*P*<.01); no substantial changes in self-reported anxiety, IBD-related QOL, and medication adherence
Katz and Leung [58], 2015	RCT	Outpatient	29	Rheum conditions	Comparison group: in-person counseling; treatment group: educational video and in-person counseling	Immediately after the intervention	Self-confidence, patient knowledge, and health care use	No difference in patient self-confidence after the intervention between the groups; trend toward increased knowledge in video group, but these results were not statistically significant; significant decrease in nurse teaching time (*P*=.01)
King et al [59], 2007	RCT	Outpatient	28	Diabetes	Comparison group: in-person counseling; treatment group: educational video, printed materials, and in-person counseling	6 weeks and 12 weeks	Disease severity and health behavior	No substantial change in HbA_1c_ level and weight 12 weeks after the intervention; significant increase in frequency of blood glucose level self-monitoring (*P*<.001) and decrease in mean postprandial blood glucose level 12 weeks after the intervention (*P*<.05)
Linné and Liedholm [60], 2006	RCT	Inpatient and outpatient	224	HF	Comparison group: none; treatment group: educational video and interactive module	2 months and 6 months	Disease severity, health care use, and knowledge	No substantial change in survival or rate of readmission 6 months after the intervention; significant increase in HF knowledge 8 weeks after the intervention (*P*=.004)
Liu et al [61], 2013	RCT	Home	59	COPD	Comparison group: printed materials and in-person counseling; treatment group: educational video and interactive module	4 months	Disease severity and QOL	Significantly improved PFTs^p^ (*P*<.05) and 6-minute walking test (*P*<.05); increased health-related QOL (*P*<.05)
Lopez-Olivo et al [62], 2020	RCT	Inpatient	221	Rheum diseases	Comparison group: printed materials; intervention group: educational video and printed materials	Immediately after the intervention, 3 months, and 6 months	Knowledge	No substantial difference in knowledge between booklet and video groups
Manns et al [63], 2005	RCT	Outpatient	62	CKD	Comparison group: in-person counseling; intervention group: educational video, quiz-based teaching, printed materials, in-person counseling, and group-based interactive session	6 months	Self-confidence	Increased intention to start self-care dialysis (*P*=.01) in intervention group
Moldofsky et al [64], 1979	RCT	Outpatient	62	Asthma	Comparison group: none; intervention group: educational video	14 to 16 months	Disease severity, medication use, health care use, and knowledge	Significant increase in asthma knowledge (*P*<.001); no substantial difference in wheezing, bronchodilator use, corticosteroid use, PCP^q^ visits, and FEV^r^
Moonaghi et al [65], 2012	RCT	Outpatient	75	CKD	Comparison group: in-person counseling; treatment group: educational video	2 weeks and 4 weeks	Self-confidence	Video noninferior to face-to-face education in confidence managing fluids and diet (*P*=.11)
Moore et al [66], 2009	RCT	Home	20	COPD	Comparison group: printed materials; treatment group: educational video, printed materials, and in-person counseling	7 to 8 weeks	Disease severity, QOL, and self-confidence	Significant increase in incremental shuttle walk test distance (*P*=.01), decrease in dyspnea (*P*=.04) and fatigue (*P*=.01), increase in emotional health (*P*<.001); no substantial effects on mastery score of the chronic respiratory disease questionnaire
Owolabi et al [67], 2019	RCT	Inpatient and outpatient	400	Hypertension	Comparison group: telephone communication; treatment group: educational video and printed materials	1, 3, 6, 9, and 12 months	Disease severity	No substantial difference in BP between comparison group and treatment group
Poureslami et al [68], 2016	RCT	Outpatient	91	COPD	Comparison group: none; treatment group: educational video	Immediately after the intervention and 3 months after the intervention	Medication use and self-confidence	Improved inhaler technique immediately after the intervention and 3 months after the intervention (*P*<.001); mixed effects on COPD management self-efficacy
Press et al [69], 2020	RCT	Inpatient and home	118	Asthma and COPD	Comparison group: in-person counseling; treatment group: educational video and interactive module	Immediately after the intervention and 1 month after the intervention	Knowledge	Interactive virtual module was noninferior to in-person teaching for inhaler technique
Ries et al [70], 1995	RCT	Outpatient	119	COPD	Comparison group: in-person counseling, exercise or physical therapy, and group discussions; intervention group: educational video, in-person counseling, and group discussions	Periodically, 2 months to 8 years	Disease severity	Video education inferior to in-person rehabilitation for treadmill endurance and shortness of breath; noninferior for FEV_1_, QOL score, and hospital days
Stromberg et al [71], 2006	RCT	Outpatient	137	HF	Comparison group: in-person counseling; treatment group: educational video, interactive module, and in-person counseling	1 month and 6 months	Health behavior, QOL, and knowledge	Significant increase in knowledge of HF definition and symptoms (*P*=.03); no substantial change in compliance with HF-recommended lifestyle or QOL
Sweat et al [72], 2001	RCT	Outpatient	2004	HIV infection	Comparison group: none; treatment group: educational video and group discussion	12 months	Health behavior and cost	Improved annualized condom use and cost savings with treatment implementation
Sweeney et al [73], 2002	RCT	Home	155	Rheum diseases	Comparison group: none; treatment group: educational video and printed materials	6 months	Disease severity, self-confidence, and health behavior	No substantial changes in daily function score, disease severity, and global well-being; significant increase in exercise self-efficacy (*P*=.045), minutes exercised (*P*=.001), and minutes spent on mobility exercise (*P*=.05)
Timmerman et al [74], 2016	RCT	Outpatient	92	Chronic pain syndromes	Comparison group: none; treatment group: educational video, printed materials, and telephone or video counseling	4 weeks and 10 weeks	Medication use, symptoms, and knowledge	No effects on medication nonadherence and mean pain intensity; improved medication knowledge (*P*<.01); decreased maximum pain intensity (*P*<.05)
Van der Palen et al [75], 1997	RCT	Outpatient	33	COPD	Comparison group: none; treatment group: in-person counseling	9 months	Medication use	Significant improvement in inhaler checklist score (*P*<.001)
Veroff et al [76], 2012	RCT	Home	480	HF	Comparison group: none; treatment group: educational video and printed materials	4 weeks and 6 months	Health behavior, QOL, disease severity, and cost	Significant increase in self-reported daily weight monitoring (*P*=03); no substantial difference in self-reported monitoring of fluid, low-sodium diet, physical activity, mental health status, and physical health; no substantial difference in claims-based cost
Tang et al [77], 2013	RCT	Home	415	Diabetes	Comparison group: none; treatment group: educational video, telephone or video counseling, nutritional counseling, and remote glucometer tracking	6 months and 12 months	Disease severity, health care use, and knowledge	Significantly improved HbA_1c_ level at 6 months (*P*<.001); no substantial difference in HbA_1c_ level at 12 months; improved LDL cholesterol level at 12 months (*P*=.001); increased diabetes knowledge (*P*=.004); no difference in BP or number of physician visits at 12 months
Wang and Chiou [78], 2011	RCT	Outpatient	60	CKD	Comparison group: none; treatment group: educational video, interactive module, and in-person counseling	4 weeks and 8 weeks	Health behavior, QOL, and knowledge	Significant increase in dialysis self-care knowledge (*P*<.001); significant increase in engagement in lifestyle choices beneficial to ESRD^s^ (*P*=.11); no significant changes in feelings of powerlessness
Williams et al [79], 2005	RCT	Outpatient	214	Diabetes	Comparison group: in-person counseling; treatment group: educational video	1 year	Disease severity	No substantial difference in reduction in HbA_1c_ levels between video and in-person groups
Wilson et al [80], 2010	RCT	Home and outpatient	435	Asthma	Comparison group: none; treatment group: educational video and printed materials	Immediately after the intervention and 1 week after the intervention	Knowledge	Significant increase in knowledge of asthma triggers (*P*<.001); improved score on assessment of inhaler use (*P*<.001)
Adarmouch et al [81], 2017	Pre-post	Home and outpatient	133	Diabetes	Educational video, interactive module, printed materials, and in-person counseling	1 month	Health behavior	Improved foot care behavior (*P*<.001)
Baldwin [82], 2013	Pre-post	Outpatient	148	CKD	Educational video	1 month	Disease severity and knowledge	Significant decrease in phosphorus level (*P*<.001); no effects on overall phosphorus knowledge
Boyde et al [83], 2012	Pre-post	Home and outpatient	38	HF	Educational video, quiz-based teaching, and printed materials	8 weeks	Health behavior and knowledge	Significant increase in HF knowledge (*P*<.001) and in HF self-care behaviors (*P*=.03)
Choy et al [84], 1999	Pre-post	Outpatient	192	Asthma	Educational video, printed materials, and in-person counseling	6 months and 12 months	Disease severity, health care use, and medication use	Significantly improved PEF (*P*<.01), FEV_1_ (*P*<.05), inhaler technique (*P*<.01), and asthma knowledge (*P*<.001); decreased hospitalizations (*P*<.01) and ED use (*P*<.001); no effect on use of oral steroids
Dubin and Rubinsky [85], 2019	Pre-post	Home	25	CKD	Educational video, interactive module, and telephone or video counseling	1 month and 2 months	Self-confidence and knowledge	Improved knowledge about CKD and dialysis (*P*<.001); improved self-efficacy in dialysis choice and care (*P*<.001)
Maslakpak and Shams [86], 2015	Pre-post	Dialysis center	167	CKD	Educational video	2 months	Disease severity and QOL	Improved QOL (*P*=.02); improved general health status (*P*=.001)
Paragas and Barcelo [87], 2019	Pre-post	Inpatient	165	Diabetes	Educational video	Immediately after intervention	Self-confidence, Knowledge	Intervention substantially improved diabetes knowledge and self-efficacy
Poole et al [88], 2013	Pre-post	Home	49	Scleroderma	Educational video and printed materials	4 to 6 weeks	Self-confidence, disease severity, QOL, and health care use	Significantly increased self-efficacy (*P*=.006); no effect on functional ability, pain, fatigue, depression, and nights spent in the hospital
Press et al [89], 2017	Pre-post	Inpatient	83	COPD and asthma	Educational video, interactive module, and quiz-based teaching	Immediately after the intervention	Medication use and self- confidence	Decrease in inhaler misuse (*P*<.001); increased confidence in inhaler use (*P*=.01)
Sadeghi et al [90], 2016	Pre-post	Inpatient	37	HF	Educational video, printed materials, and in-person counseling	6 months	Health behavior	Increased rate of POLST^t^ form completion (*P*=.03); no effect on completion of advance directive
Short [91], 2019	Pre-post	Outpatient	15	Chronic pain (migraine)	Educational video and in-person counseling	8 weeks	Disease severity, self-confidence, symptoms, and medication use	Decreased disease severity (*P*=.04); increased self-efficacy (*P*=.01); decreased symptoms (*P*=.002); decreased medication use (*P*=.005)
Smith et al [92], 2005	Pre-post	Home	10	HF	Educational video and telephone or video counseling	60 days	Knowledge	Improved HF knowledge recall (*P* values not reported)
Sobel et al [93], 2009	Pre-post	Outpatient	130	Asthma	Educational video	Immediately after the intervention	Knowledge	Significant increase in asthma knowledge (*P*<.001)

^a^RCT: randomized controlled trial.

^b^HF: heart failure.

^c^QOL: quality of life.

^d^COPD: chronic obstructive pulmonary disease.

^e^HbA_1c_: glycated hemoglobin.

^f^CKD: chronic kidney disease.

^g^ED: emergency department.

^h^PEF: peak expiratory flow.

^i^NR: not reported.

^j^BP: blood pressure.

^k^CVD: cardiovascular disease.

^l^LDL: low-density lipoprotein.

^m^FEV_1_: forced expiratory volume in 1 second.

^n^BDI: Beck Depression Inventory.

^o^IBD: inflammatory bowel disease.

^p^PFT: pulmonary function test.

^q^PCP: primary care provider.

^r^FEV: forced expiratory volume.

^s^ESRD: end-stage renal disease.

^t^POLST: physician orders for life-sustaining treatment.

### General Characteristics of the Reviewed Papers

The included studies’ publication years ranged from 1979 to 2021. Two-thirds (40/59, 68%) of the studies were conducted in North American or European countries, and 31% (18/59) took place in an urban setting. A majority of the studies (39/59, 66%) were superiority RCTs, 22% (13/59) were pre-post studies, and 12% (7/59) were noninferiority RCTs. The most commonly represented chronic diseases included diabetes (11/59, 19%) [36,39,49-52,59,77,79,81,87], asthma (11/59, 19%) [43,44,46,55,64,69,80,84,89,93], COPD (8/59, 14%) [38,61,66,68-70,75,89], and heart failure (9/59, 15%) [35,38,41,45,60,71,76,83,90]. Less commonly represented diseases included CKD (7/59, 12%), rheumatologic diseases (5/59, 8%), hypertension (5/59, 8%), chronic pain syndromes (4/59, 7%), HIV infection (2/59, 3%), and IBD (1/59, 2%).

Of the 59 studies, 22 (37%) explicitly targeted a particular group with the intervention, such as older adults, veterans, and Black patients. Among the studies that reported overall sample demographics (30/59, 51%), the mean age was 53.9 (SD 10.6) years, 52.5% of the participants were male, and 47.5% were female. The majority of the studies (45/59, 76%) did not include race data for the overall sample. Approximately one-third (20/59, 34%) of the studies explicitly stated a conceptual framework for the interventions (Table 2), including adult learning theory [41,67,69,83,87,89,91], goal management theory [51,62,77], and health literacy perspective [64,93]. In the included studies, the median number of participants enrolled was 130 (IQR 65-199), and the median number of participants included in analysis was 119 (IQR 60-167). Only 3 (5%) of the 59 studies reported a sample mainly composed of individuals with low socioeconomic status [54,84,92]. A little more than half (32/59, 54%) of the interventions took place in the outpatient setting, 40% (23/59) took place at home, 19% (11/59) in the inpatient setting, and 3% (2/59) in the emergency department (ED).

All interventions included an educational video, and many of the interventions included printed materials (24/59, 41%), in-person counseling (19/59, 32%), and interactive modules (12/59, 20%; Table 3).

The plurality of video-based interventions (28/59, 47%) occurred once (Table 4). A quarter (16/59, 27%) of the interventions took place at the patients’ discretion (Table 4).

**Table 2:** Extracted studies: conceptual framework and demographics (n=59).

Studies	Conceptual framework	Age (years), mean (SD)	Male (%)	White (%)	Black (%)
Albert et al [35], 2007	Unknown	—^a^	—	—	—
Albikawi et al [36], 2016	Self-efficacy theory	51 (7)	46	N/A^b^	N/A
Allam et al [37], 2015	Unknown	58 (12)	54	—	—
Barker et al [38], 2020	Unknown	69 (11)	95	—	—
Bell et al [39], 2012	Unknown	58 (11)	55	31	58
Boulware et al [40], 2013	Unknown	—	—	—	—
Boyde et al [41], 2018	Adult learning theory	64 (12)	73	—	—
Calderon et al [42], 2009	Unknown	30.4 (9)	61.6	—	—
Chen et al [43], 2010	Self-efficacy theory	—	—	N/A	N/A
Cordina et al [44], 2001	Unknown	43.2 (180)	50.6	—	—
Dilles et al [45], 2011	Unknown	72.8 (11)	70.2	—	—
Ebrahimabadi et al [46], 2018	Unknown	—	—	N/A	N/A
Elander et al [47], 2011	Taxonomy of behavior change techniques	—	—	—	—
Emmett et al [48], 2005	Unknown	—	—	—	—
Gerber et al [49], 2005	Conditions of learning	—	—	—	—
Glasgow et al [50], 1996	Unknown	—	—	—	—
Glasgow et al [51], 2009	Goal management theory	—	—	—	—
Gravely et al [52] (2011)	Unknown	54.3 (NR^c^)	—	—	13
Hickman et al [53], 2015	Unknown	—	—	—	—
Houston et al [54], 2017	Narrative theory	—	92	0	100
Huang et al [55], 2008	Unknown	—	—	N/A	N/A
Jerjes et al [56] 2007	Unknown	37 (13)	11	—	—
Kamat et al [57], 2018	Unknown	37 (11)	73	N/A	N/A
Katz and Leung [58], 2015	Unknown	—	—	—	—
King et al [59], 2007	Unknown	58.6 (10)	61	—	—
Linné and Liedholm [60], 2006	Unknown	—	—	—	—
Liu et al [61], 2013	Unknown	—	—	N/A	N/A
Lopez-Olivo et al [62], 2020	Goal management theory	50.8 (13)	15	20	20
Manns et al [63], 2005	Unknown	64.4 (NR)	—	—	—
Moldofsky et al [64], 1979	Health literacy perspective	46 (NR)	—	—	—
Moonaghi et al [65], 2012	Unknown	49.8 (12)	60	N/A	N/A
Moore et al [66], 2009	Unknown	—	—	—	—
Owolabi et al [67], 2019	Adult learning theory	57 (12)	63.5	—	—
Poureslami et al [68], 2016	Community-based participatory approach	—	78	—	—
Press et al [69], 2020	Adult learning theory	54.5 (13)	36	3	97
Ries et al [70], 1995	Unknown	—	73	—	—
Stromberg et al [71], 2006	Unknown	—	—	—	—
Sweat et al [72], 2001	Unknown	—	—	—	—
Sweeney et al [73], 2002	Unknown	—	—	—	—
Timmerman et al [74], 2016	Unknown	—	—	—	—
Van der Palen et al [75], 1997	Unknown	55 (9)	64	—	—
Veroff et al [76], 2012	Unknown	—	—	—	—
Tang et al [77], 2013	Goal management theory	—	—	—	—
Wang and Chiou [78], 2011	Unknown	—	—	N/A	N/A
Williams et al [79], 2005	Unknown	—	—	—	—
Wilson et al [80], 2010	Unknown	53.3 (10)	43	34.9	50.8
Adarmouch et al [81] (2017)	Unknown	55 (11)	33.8	—	—
Baldwin [82], 2013	Unknown	—	—	—	—
Boyde et al [83], 2012	Adult learning theory	—	71	—	—
Choy et al [84], 1999	Unknown	48.6 (NR)	39.1	N/A	N/A
Dubin and Rubinsky [85], 2019	Unknown	65 (15)	68	48	20
Maslakpak and Shams [86], 2015	Unknown	—	—	N/A	N/A
Paragas and Barcelo [87], 2019	Adult learning theory	—	—	N/A	N/A
Poole et al [88], 2013	Self-efficacy theory	53.9 (13)	8	82	8
Press et al [89], 2017	Adult learning theory	47.9 (14)	37.8	6	94.4
Sadeghi et al [90], 2016	Unknown	70.6 (12)	51	—	—
Short [91], 2019	Adult learning theory	40.9 (NR)	6.7	100	0
Smith et al [92], 2005	Model of health behavior and family care theory	67 (NR)	60	100	0
Sobel et al [93], 2009	Health literacy perspective	50.2 (15)	23.8	0	100

^a^Not available.

^b^N/A: not applicable.

^c^NR: not reported.

**Table 3:** Intervention components (n=59).

	Intervention, n (%)	Control, n (%)
Educational video	59 (100)	4 (7)
Printed materials	24 (41)	11 (19)
In-person counseling	19 (32)	19 (32)
Interactive module	12 (20)	1 (2)
Telephone or video counseling	8 (14)	0 (0)
Quiz-based teaching	6 (10)	1 (2)
Nutritional counseling	2 (3)	0 (0)
Game	1 (2)	0 (0)
Exercise or physical therapy	0 (0)	1 (2)
None	0 (0)	24 (41)

**Table 4:** Intervention frequency (n=59).

	Intervention, n (%)	Control, n (%)
Once	28 (47)	19 (32)
At select time intervals	20 (34)	7 (12)
At patient’s discretion	16 (27)	8 (14)
None	0 (0)	25 (42)

### Outcomes

Half (132/262, 50.4%) of the metrics were outcome measures and half (130/262, 49.6%) were process measures. Outcome measures included disease severity, health care use, QOL, symptoms, and cost. Process measures included patient knowledge, health behavior, self-efficacy, and medication use. A meta-analysis was not performed because the interventions were too heterogeneous, varying in setting, frequency, and design. Most of the interventions (53/59, 90%) had at least 1 successful or noninferior component. Studies that explicitly stated conceptual frameworks did not have more substantial improved outcomes.

### Outcome Measures

#### Disease Severity

The most common outcome measured (29/59, 48%) was disease severity [35,38,39,44,48-51,53-56,59-61,64,66,67,70,73,74,76,​^77^,^79^,^82^,^84^,^88^,^91^]. The specific measures of disease severity included blood pressure [48,49,51,53,54,67,77], glycated hemoglobin (HbA_1c_) level [39,49-51,59,77], pulmonary function tests [44,55,64,84], and self-reported patient scoring of disease severity [35,38,44,56,67,70,73,84]. Among the studies that included at least 1 disease severity measure (29/59, 48%), a total of 69 outcomes were captured. The majority of disease severity measures were for diabetes (20/69, 29%) [39,49-51,59,77,79], COPD (16/69, 23%) [61,66,70], and asthma (12/69, 17%) [44,55,64,84,94]. Of the 69 measures, 45 (65%) were reported from superiority RCTs, followed by 14 (20%) from noninferiority RCTs and 10 (15%) from pre-post studies. The plurality of comparison groups (29/69, 42%) had no intervention, but some components of comparison groups included in-person counseling (16/69, 23%) or printed materials (10/69, 15%). All intervention groups had video-based education components, and many of them had additional components, including printed materials (36/69, 52%), in-person counseling (27/69, 39%), and interactive modules (12/69, 17%). Although 43% (30/69) of the disease severity outcomes favored the intervention group in raw difference, 33% (23/69) of the treatment groups had statistically significantly improved outcomes, and 12% (8/69) of the video-based interventions yielded noninferior outcomes. Of the 69 measured disease severity outcomes, 38 (55%) were not substantially affected by video-based interventions. Successful disease severity outcomes included pulmonary function tests, the 6-minute walk test in patients with COPD, phosphorus level in patients with CKD, HbA_1c_ level, low-density lipoprotein cholesterol level, and blood pressure [35,39,44,49-51,55,61,66,67,77,82,84]. Some of the disease severity outcomes that showed no improvement after video-based interventions (38/69, 55%) included ankylosing spondylitis daily function scores, 10-year cardiovascular disease risk changes, temporomandibular joint disability scores, and rheumatologic functional ability and pain scales [38,39,44,48-50,53,54,56,59,60,66,70,73,76,77,84,88,94].

#### Health Care Use

Of the 59 studies, 18 (31%) involved at least 1 measure of health care use, capturing 28 individual measures [35,37,38,41,43,44,48,55,57,58,60,63,64,70,77,84,88,91]. These measures included hospital admissions (7/28, 25%) [41,44,55,60,70,84,88], primary care visits (4/28, 14%) [43,58,62,64,84], and ED use (4/28, 14%) [35,55,84,91]. Diseases of interest included asthma (7/28, 25%) [43,44,55,64,84], COPD (6/28, 21%) [38,70], heart failure (6/28, 21%) [35,41,60], and rheumatologic disorders (3/28, 11%) [37,58,88]. Of the 28 measures, 17 (61%) were drawn from superiority RCTs, 6 (21%) from noninferiority RCTs, and 5 (18%) from pre-post trials. Comparison groups involved no intervention (13/28, 46%), printed materials (10/28, 36%), and in-person counseling (9/28, 32%). All intervention groups of interest included educational videos, and the majority involved printed materials (19/28, 68%) and in-person counseling (13/28, 46%) as well. Of the 28 outcomes measured, 11 (39%) showed improvement (including decreased unplanned use of health care services and increased compliance with scheduled health care visits) [44,54,57,84] with video-based interventions, and 7% (n=2) showed noninferior outcomes. In 50% (14/28) of the health care use outcomes, the video-based intervention had no effect.

#### QOL and Social-Emotional Well-Being

Of the 59 studies, 17 (29%) involved at least 1 measure of QOL or social-emotional well-being for a total of 26 discrete measures [36,44,47,50,56,57,61,64,69-71,73,76,78,86,88]. Most of the represented diseases of interest included asthma (7/26, 27%) [44,64], COPD (5/26, 19%) [61,69,70], and IBD (5/26, 19%) [57]. The measures included self-reported depression [56,57,88], anxiety [57], and QOL survey scales [36,44,50,57,​^61^,^70^,^71^,^73^,^86^]. The majority of the papers assessing QOL and well-being were superiority RCTs (13/17, 76%). The majority of the comparison groups (10/17, 59%) included no intervention. All interventions of interest included a video-based component, and some of them included printed materials (6/17, 35%), in-person counseling (5/17, 29%), and interactive modules (4/17, 24%). The majority of the interventions (21/26, 81%) did not have a substantial effect on QOL and well-being outcomes.

#### Symptoms

Of the 59 studies, 4 (7%) reported a total of 7 symptom outcomes [64,74,86,91]. The represented diseases of interest were chronic pain syndromes [74,91], asthma [64], and CKD [86], measuring pain intensity, wheezing, and CKD symptom frequency, respectively. The intervention improved outcomes in only 2 (29%) of the 7 cases.

#### Cost

Of the 59 studies, 2 (3%) reported cost outcomes [72,76]. One study measured cost savings from an HIV infection intervention, and the other measured health care claims incurred after a heart failure intervention. The heart failure intervention found no substantial effect on cost [76]. The study of an HIV infection video intervention found that it would be a cost-effective intervention for clients of a clinic for male individuals with sexually transmitted infection considered high risk [72].

### Process Measures

#### Patient Knowledge

After disease severity, patient knowledge was the most frequently reported measure. Of the 59 studies, 24 (41%) had at least 1 knowledge component among their outcomes [41,42,44,45,49,52,55,60,62,64,69,71,74,77,78,80,82-85,87,92,93]. Knowledge outcomes were mainly composed of disease-specific questionnaires about symptoms, treatment, management, and pathophysiology. A total of 40 knowledge outcomes were extracted. Of these 40 measures, 16 (40%) involved patients with asthma [44,55,64,80,84,93], and 7 (18%) involved patients with congestive heart failure [41,45,60,71,92]. Of the 40 knowledge outcomes, 25 (63%) were drawn from superiority RCTs, 12 (30%) from pre-post trials, and 3 (8%) from noninferiority RCTs. The majority (23/40, 58%) had a comparator group with no intervention. Other comparator groups involved in-person counseling (9/40, 23%) and printed materials (8/40, 20%). In addition to educational videos, the intervention components included printed materials (12/40, 30%), interactive modules (8/40, 20%), and in-person counseling (7/40, 18%). The majority of the knowledge outcomes (32/40, 80%) contained a component of knowledge about disease management skills. Video-based intervention improved knowledge in 75% (30/40) of the outcomes.

#### Health Behavior

Approximately one-third (20/59, 34%) of the studies measured at least 1 health behavior [35,37,40,41,43,45,47,50,51,55,59,​^70^-^73^,^76^,^78^,^81^,^83^,^90^]. Among these 20 studies, 38 total outcomes were measured. The majority of the measures (26/38, 68%) were self-reported behavior scales, including exercise, diet, blood glucose self-monitoring, and condom use. Of the 38 measures, 15 (39%) were for patients with congestive heart failure [35,41,45,71,76], 8 (21%) for patients with diabetes [50,51,59,81], and 7 (19%) for patients with asthma [43,55]. Among the comparator groups, 42% (16/38) involved no intervention, whereas 13% (5/38) involved printed materials. All intervention groups involved an educational video, and many of them (31/38, 82%) involved printed materials as well, whereas 26% (10/38) involved interactive modules. The most common health behavior measures were various self-care scales (13/38, 34%) [35,41,45,55,78,81], physical activity (6/38, 16%) [37,43,51,73,76], and fluid or diet monitoring (6/38, 16%) [50,51,71,76]. Of the 38 health behavior outcomes, 21 (55%) were significantly improved by the video-based intervention. Half (4/8, 50%) of the health behavior outcomes from noninferiority trials showed noninferior outcomes compared with more involved in-person interventions.

#### Self-Confidence

Of the 59 studies, 17 (29%) measured patient self-confidence outcomes, including self-efficacy in performing health behaviors and managing symptoms, using measures such as the insulin management self-efficacy scale and self-efficacy scale for adult asthmatic patients [37,41,43,49-51,55,56,58,65,68,73,83,​^85^,^87^-^89^]. A total of 23 measures were captured, including 6 (26%) involving patients with rheumatologic disorders [37,58,73,88], 6 (26%) involving patients with diabetes [49,51,87], and 4 (17%) involving patients with asthma [43,55,69,89]. All interventions of interest involved educational videos, and many of them included printed materials (9/23, 39%) and interactive modules (5/23, 22%). Authors reported improved self-confidence in 52% (12/23) of the outcomes, noninferiority in 9% (2/23), and no effect in 30% (7/23).

#### Medication Use

Of the 59 studies, 14 (24%) measured medication use, often level of adherence to prescribed medications [43,44,46,51,54,57,74] and correct inhaler technique [68,75,89]. Across the 14 studies, 29 measures of medication use were captured [37,39,43,44,46,51,54,57,64,68,74,75,89,91]. The most common diseases of interest were asthma (13/29, 45%) [43,44,46,64,69,89] and COPD (12, 43%) [68,69,75,89]. The majority of the measures (20/29, 69%) were from RCTs. All interventions of interest included educational videos, and some of them included interactive modules (7/29, 24%), in-person counseling (7/29, 24%), and quiz-based teaching (6/29, 21%). Of the 29 medication use outcomes, 14 (48%) showed improved outcomes among the video-based intervention group. For a little more than half (15/29, 52%) of the outcomes, authors concluded that the intervention improved or yielded noninferior outcomes.

### Noninferiority Trials

Of the 59 studies, 7 (12%) studied noninferiority of video-based tools compared with in-person education [38,42,51,65,67,69,70]. Among these papers, 82% (28/34) of the outcomes resulting from video-based tools were noninferior, 6% (2/34) resulted in inferior outcomes, and 12% (4/34) resulted in mixed or unclear effects.

### Effects of Repeated Intervention

A little more than half (31/59, 53%) of the studies involved repetition of the intervention. Outcome evaluation ranged from immediately after the intervention to 16 months after the intervention. The majority of the papers (28/31, 90%) assessed outcomes that occurred on a short-term basis (within 1 year). Outcomes resulting from repetition of the intervention were more frequently improved than those resulting from the intervention completed once (49% vs 40%, respectively; *P*=.23).

### Quality and Bias

The RoB 2 (Multimedia Appendix 2 [35-80]) was used to evaluate the RCTs (n=46). Of the 46 RCTs, 24 (57%) had low risk of overall bias, 17 (37%) had some concerns for risk of overall bias, and 5 (14%) had high risk of overall bias. Of the 46 RCTs, 15 (33%) carried risk of bias owing to the lack of blinding of outcomes assessors, 9 (20%) had risk for bias owing to problems with randomization, and 2 (4%) carried risk of bias owing to loss of a substantial number of study participants over the study period. The ROBINS-I tool (Multimedia Appendix 3 [81-93]) was used to evaluate the pre-post trials (n=13). Of these 13 trials, 6 (46%) were judged to have low risk of bias and 7 (54%) had moderate risk of bias. Risk of bias occurred because of risk of confounding, missing data, and outcome assessors’ awareness of participant group assignment.

## Discussion

### Principal Findings

A systematic review of the efficacy of video-based education across major chronic illnesses has not previously been performed. This study confirms prior published evidence that video-based tools improve patient knowledge in the short term. We extend past findings by demonstrating that there is less consistent evidence for the effectiveness of these tools in improving patients’ QOL and disease severity [96,97]. Furthermore, our findings confirm a prior study that demonstrated a lack of uniform evidence of significant improvement of health behaviors from video-based educational interventions [98]. Prior reviews focused on comparing video-based interventions with either in-person counseling or printed materials. Our study demonstrates how health interventions have become more complex, involving several interventions alongside videos, including printed materials, interactive modules, and telephone or SMS text message communication.

### Patient Knowledge Outcomes

Video-based interventions were most successful in improving knowledge measures; for instance, the majority of patient knowledge outcomes (eg, Dutch Heart Failure Knowledge Scale score, diabetic foot care knowledge, knowledge of asthma triggers, and inhaler technique knowledge) [44,52,60,62,69,71,74,77,78,80,83-85,87,91-93] showed significant improvements after the video-based intervention. This is consistent with prior literature [96]. Furthermore, a little more than half of the self-efficacy outcomes were improved by video-based interventions. Examples of successful self-efficacy outcomes included pain self-efficacy scales, asthma attack prevention self-efficacy surveys, inhaler technique confidence, and heart failure self-care confidence scales [43,49,73,83,85,87-89,91]. Some of the studies explicitly stated self-efficacy theory as a framework in tool development, whereas many of the others included patient empowerment language and disease self-management techniques in their tools [36,43,86,88]. These findings suggest that video-based education tools with goals of increasing patient knowledge are more likely to be successful. This systematic review showed a trend toward increased success in video-based educational tools with repeated exposure to the intervention, but these results were not statistically significant.

### Health Behavior and Medication Use Outcomes

Ideally, increases in patients’ knowledge and self-efficacy lead to increased engagement in positive health behaviors and appropriate medication use. A little more than half of the health behavior outcomes, such as exercise, diet, and health self-monitoring, were improved by video-based intervention. Examples of successful health behavior outcomes included diabetes foot care behavior; heart failure management behaviors such as daily weight, fluid, and salt monitoring; physical activity; caloric fat intake reduction; and blood glucose self-monitoring [35,40,43,47,50,59,72,73,76,78,81,83,90]. A third of the included papers measured at least 1 health behavior result, indicating that there is active interest in how video-based interventions modify behaviors. Our review demonstrated that only half of the medication use outcomes were improved by video-based intervention. The majority of successful medication use outcomes were inhaler use and technique [43,51,68,75,89]. Most of the unsuccessful medication use outcomes were for pain medication use measures in IBD and chronic pain syndromes [37,57,74]. Although it is promising that some successful health behavior and medication use outcomes were achieved through video-based education tools, these findings suggest that these tools alone may be insufficient to consistently improve these outcomes in practice.

It is not surprising that educational interventions more consistently improve patient knowledge rather than behaviors because several factors beyond education may influence a patient’s ability to engage in health behaviors or adhere to medications. Examples include community resources, socioeconomic status, social support, and stress. These findings suggest that video-based educational tools have the potential to improve health behaviors and medication use but may need to be augmented with additional support to consistently make positive health behavior changes accessible to patients.

### Patient Health and Cost Outcomes

Ultimately, the interventions aimed to increase patient engagement in positive health behaviors, and we hypothesized that this engagement would result in reduced disease severity, fewer symptoms, improved QOL, and decreased inappropriate or cost-ineffective health care use. Increased patient engagement has been shown to improve health-related outcomes, including clinical indicators and avoidance of costly use [20,99]. Our systematic review demonstrated that video-based education tools less consistently improved disease severity and cost, extending the findings of a prior systematic review [98]. Disease severity was the most frequently reported outcome, but only one-third of the disease severity outcomes were improved by video-based interventions, predominantly in COPD, diabetes, and hypertension. Ankylosing spondylitis and other rheumatologic function scores and temporomandibular joint disability scores were not improved with video-based interventions. Health care use improvement was defined as increased adherence to scheduled follow-up visits and decreases in unplanned hospital readmissions and ED use. Among the included papers, a minority of health care use outcomes showed significant improvement. More than three-quarters of the QOL outcomes, such as depression and anxiety scales, self-rated QOL scales, and global well-being scores, were not substantial improved by video-based tools. These findings suggest that video-based tools do not consistently improve patients’ disease severity, health care use, and QOL or, alternatively, that interventions and study follow-up must extend over longer periods of time to measure changes in these outcomes.

Of all the measures of interest, cost was the most underrepresented. Only 2 (3%) of the 59 papers reported the effect of video-based interventions on cost measures at all. This may be due to a requirement of longer follow-ups, larger sample sizes, and more complex data collection required to demonstrate cost effects. Cost measures may be indirectly measured through health care use variables such as adherence to preventive and scheduled health care encounters and rates of unscheduled ED visits and hospitalizations. One opportunity for further study includes continuing data collection through longer follow-up periods to clarify the effects of these interventions, if any, on cost.

### Diversity of Video-Based Interventions

There is great diversity in both the chronic health conditions studied in the literature and the specific educational programming offered. Often, interventions combined videos with interactive modules, SMS text message reminders, and telephone counseling. These combinations of interventions may simulate the diverse sources of information that patients are exposed to in practice, but they complicate analyses to determine what programming components specifically contribute to an improvement in patients’ knowledge, confidence, behaviors, and outcomes. Virtual platforms and technologies continue to be leveraged to augment traditional health care delivery and patient education tools [100-104]. Although only a few of the papers studied noninferiority of video-based education tools to in-person education, the majority of these papers found at least 1 noninferior outcome, indicating that video-based tools are promising compared with in-person strategies. Of the 59 included studies, only 1 (2%) directly compared video-based intervention with other virtual education interventions, comparing video-based education with SMS text message communications, and found improved outcomes with texting communications [67]. Further clarification of the relative efficacies of these tools is important as televisits, video visits, and patient portals become more prevalent. Furthermore, assessment of new technology must take into consideration variations in access to, as well as knowledge of, and dexterity with, technology in patient populations [22,105,106].

### Conceptual Frameworks of Video-Based Educational Tools

A minority of the included studies (20/59, 34%) explicitly stated the conceptual frameworks underlying their video-based educational tool. This limited the analysis of which conceptual frameworks, including adult learning theory, goal management theory, health literacy perspective, self-efficacy theory, and community-based participatory research, yielded more successful outcomes. Further study is needed to clarify which educational theories and specific components make video-based patient education tools most successful.

### Study Limitations

Although this study used rigorous methods to review the existing literature, there are important limitations. First, the heterogeneity of the factors reviewed, including large number of chronic illnesses, specific programming components, timing, and intervention goals, makes drawing specific conclusions about the efficacy of video-based tools difficult and precludes execution of a meta-analysis. In addition, enrollment in studies and engagement with video-based health tools in trials may not reflect the *real-world* behaviors of the population at large. Patients who enroll in research studies tend to have higher health literacy, and the effect of varying health literacy on video- and technology-based health tools will need to be assessed to ensure equity as the use of these tools continues [107-109]. A minority of the studies targeted specific populations, such as those with lower health literacy, and none targeted patients with low eHealth literacy. Strategies of studies targeting specific populations with low health literacy included nontechnical language, narrative testimonials, and inclusion of target populations in the development of the educational video-based tools [49,54,68]. Studying these populations is vital in understanding the true efficacy of video-based tools for all patients [22].

### Conclusions

This study suggests that for many process measures and some outcome measures, video-based chronic illness management tools are effective in improving patient outcomes. In some of the cases, video-based tools were found to be superior to printed materials and noninferior to in-person counseling. From time constraints with regard to clinic visits to changing policies on social distancing as the COVID-19 pandemic evolves, a need for alternatives and adjuncts to in-person counseling is clear. Patient-facing video-based tools for chronic illness management show promise. Additional studies that assess disease severity, symptoms, and cost measures over longer follow-up periods and specifically targeting patients along the spectrum of digital competencies are needed to understand how to optimize patients’ benefit from video-based education tools.

## Appendix

Multimedia Appendix 1 Final search terms for PubMed and CINAHL.

Multimedia Appendix 2 Results of the risk-of-bias evaluation conducted using the revised Cochrane risk-of-bias tool for randomized trials.

Multimedia Appendix 3 Results of the risk-of-bias evaluation conducted using the Risk of Bias in Nonrandomized Studies of Interventions tool.

## Abbreviations

CKD: chronic kidney disease
COPD: chronic obstructive pulmonary disease
ED: emergency department
HbA1c: glycated hemoglobin
IBD: inflammatory bowel disease
MeSH: Medical Subject Headings
PRISMA: Preferred Reporting Items for Systematic Reviews and Meta-Analyses
QOL: quality of life
RCT: randomized controlled trial
REDCap: Research Electronic Data Capture
RoB 2: revised Cochrane risk-of-bias tool for randomized trials
ROBINS-I: Risk of Bias in Nonrandomized Studies of Interventions

## Data Availability

As the data used in this manuscript are highly granular and contain potentially sensitive patient information, public sharing of the data would be considered a breach of the protocol requirements of the University of Chicago institutional review board. Interested researchers may contact the corresponding author for data requests.
