# Supplementary material for: Video-Based Educational Interventions for Patients With Chronic Illnesses: Systematic Review
Source: J Med Internet Res. 2023 Jul 19;25:e41092. doi: 10.2196/41092 (PMC10398560; doi:10.2196/41092)
Supplement: Multimedia Appendix 1 [file jmir_v25i1e41092_app1.docx]

**Multimedia Appendix 1**

Final search terms for PubMed and CINAHL.

**PubMed:**

((patient education) AND (((((("audiovisual"[All Fields] AND "aids"[All Fields]) OR "audiovisual aids"[All Fields] OR ("audiovisual"[All Fields] AND "aid"[All Fields]) OR "audiovisual aid"[All Fields])) OR ((("Audiovisual Aids"[Mesh:NoExp]) OR "Videodisc Recording"[Mesh]) OR "Videotape Recording"[Mesh])) OR video OR DVD OR CD-ROM))) AND ((((re-hospitalizations OR re-hospitalization OR readmissions))) OR ((patient discharge OR self-care OR Patient Readmission)))

**CINAHL:**

1. Self-management and video and adult
2. Self-monitoring and video
3. Self-regulation and video
4. Self-care and video and adult
