# Supplementary material for: Video-Based Educational Interventions for Patients With Chronic Illnesses: Systematic Review
Source: J Med Internet Res. 2023 Jul 19;25:e41092. doi: 10.2196/41092 (PMC10398560; doi:10.2196/41092)
Supplement: Multimedia Appendix 2 [file jmir_v25i1e41092_app2.docx]

**Multimedia Appendix 2**

Risk of Bias (ROB2) Results

| Reference | Randomization Process | Deviation from Intended Intervention | Missing Outcome Data | Bias in Measurement | Selection of Reported Result | Overall Risk of Bias |
| --- | --- | --- | --- | --- | --- | --- |
| Kamat[57] | Some concerns | Low risk | Low risk | Low risk | Some concerns | Some concerns |
| Boyde[41] | Low risk | Low risk | Low risk | Low risk | Low risk | Low risk |
| Houston[54] | Low risk | Low risk | Low risk | Some concerns | Low risk | Some concerns |
| Poureslami[68] | Low risk | Low risk | Low risk | Low risk | Low risk | Low risk |
| Timmerman[74] | Low risk | Low risk | Low risk | Low risk | Low risk | Low risk |
| Albikawi[36] | Low risk | Low risk | Low risk | Low risk | Low risk | Low risk |
| Hickman[53] | Low risk | Low risk | Low risk | Low risk | Low risk | Low risk |
| Allam[37] | Low risk | Low risk | Low risk | Low risk | Low risk | Low risk |
| Katz[58] | High risk | Low risk | Low risk | High risk | Low risk | High risk |
| Liu[61] | Low risk | Low risk | Low risk | Low risk | Low risk | Low risk |
| Moonaghi[65] | Low risk | Low risk | Low risk | Low risk | Low risk | Low risk |
| Boulware[40] | Low risk | Low risk | Low risk | Low risk | Low risk | Low risk |
| Bell[39] | Low risk | Low risk | Low risk | Low risk | Low risk | Low risk |
| Veroff[76] | Low risk | Low risk | Low risk | Low risk | Low risk | Low risk |
| Gravely[52] | High risk | Low risk | Low risk | Low risk | Low risk | High risk |
| Elander[47] | Low risk | Low risk | Some concerns | Low risk | Low risk | Some concerns |
| Chen[43] | Some concerns | Low risk | Low risk | Low risk | Low risk | Some concerns |
| Wilson[80] | Low risk | Low risk | Low risk | Some concerns | Low risk | Some concerns |
| Glasgow[50] | Low risk | Low risk | Low risk | Some concerns | Low risk | Some concerns |
| Calderon[42] | Low risk | Low risk | Low risk | Some concerns | Low risk | Some concerns |
| Moore[66] | Low risk | Low risk | Low risk | Some concerns | Low risk | Some concerns |
| Huang[55] | Low risk | Low risk | Low risk | Low risk | Low risk | Low risk |
| Albert[35] | Low risk | Low risk | Low risk | Low risk | Low risk | Low risk |
| King[59] | Low risk | Low risk | Low risk | Some concerns | Low risk | Some concerns |
| Linne[60] | Low risk | Low risk | Low risk | Low risk | Low risk | Low risk |
| Stromberg[71] | Low risk | Low risk | Low risk | Low risk | Low risk | Low risk |
| Manns[63] | Low risk | Low risk | Low risk | Low risk | Low risk | Low risk |
| Emmett[48] | Low risk | Low risk | Low risk | Low risk | Low risk | Low risk |
| Gerber[49] | Low risk | Low risk | Low risk | Some concerns | Low risk | Some concerns |
| Williams[79] | Low risk | Low risk | Low risk | Low risk | Low risk | Low risk |
| Sweeney[73] | Low risk | Low risk | Low risk | Some concerns | Low risk | Some concerns |
| Cordina[44] | Some concerns | Low risk | Low risk | Some concerns | Low risk | Some concerns |
| Sweat[72] | Some concerns | Low risk | Low risk | Low risk | Low risk | Some concerns |
| van der Palen[75] | Low risk | Low risk | Low risk | Some concerns | Low risk | Some concerns |
| Glasgow [51] | Low risk | Low risk | Low risk | Low risk | Low risk | Low risk |
| Ries[70] | Low risk | Low risk | Low risk | Low risk | Low risk | Low risk |
| Moldofsky[64] | Low risk | Low risk | Low risk | Some concerns | Low risk | Some concerns |
| Lopez-Olivo[62] | Low risk | Low risk | Some concerns | Some concerns | Low risk | Some concerns |
| Barker[38] | Low risk | Low risk | Low risk | Low risk | Low risk | Low risk |
| Press[69] | Low risk | Low risk | Low risk | Low risk | Low risk | Low risk |
| Ebrahimabadi[46] | Low risk | Low risk | Low risk | Some concerns | Low risk | Some concerns |
| Owolabi[67] | Low risk | Low risk | Low risk | Low risk | Low risk | Low risk |
| Tang[77] | Low risk | Low risk | Low risk | Low risk | Low risk | Low risk |
| Wang[78] | High risk | Low risk | Low risk | Low risk | Low risk | High risk |
| Dilles[45] | High risk | Low risk | Low risk | Some concerns | Low risk | High risk |
| Jerjes[56] | High risk | Low risk | Low risk | Low risk | Low risk | High risk |
