# Supplementary material for: Video-Based Educational Interventions for Patients With Chronic Illnesses: Systematic Review
Source: J Med Internet Res. 2023 Jul 19;25:e41092. doi: 10.2196/41092 (PMC10398560; doi:10.2196/41092)
Supplement: Multimedia Appendix 3 [file jmir_v25i1e41092_app3.docx]

**Multimedia Appendix 3**

Risk of Bias ROBINS-I Results

| Reference | Risk due to Confounding | Risk due to Selection of Participants | Risk due to Classification of Intervention | Risk due to Deviation from Intervention | Risk due to Missing Data | Risk due to Measurement of Outcome | Risk due to Selection of Reported Result | Overall Risk of Bias |
| --- | --- | --- | --- | --- | --- | --- | --- | --- |
| Adarmouch[81] | Low | Low | Low | Low | Low | Low | Moderate | Moderate |
| Press[89] | Low | Low | Low | Low | Low | Low | Low | Low |
| Sadeghi[90] | Low | Low | Low | Low | Low | Low | Low | Low |
| Poole[88] | Low | Low | Low | Low | Low | Low | Low | Low |
| Baldwin[82] | Low | Low | Low | Low | Moderate | Low | Low | Moderate |
| Boyde[83] | Low | Low | Low | Low | Low | Low | Low | Low |
| Sobel[93] | Low | Low | Low | Low | Low | Low | Low | Low |
| Smith[92] | Low | Low | Low | Low | Low | Moderate | Low | Moderate |
| Choy[84] | Low | Low | Low | Low | Moderate | Low | Low | Moderate |
| Paragas[87] | Moderate | Moderate | Low | Low | Low | Low | Low | Moderate |
| Short[91] | Low | Low | Low | Low | Low | Low | Moderate | Moderate |
| Dubin[85] | Moderate | Low | Low | Low | Low | Low | Low | Moderate |
| Maslakpak[86] | Low | Low | Low | Low | Low | Low | Low | Low |
